# Supplementary material for: Hierarchical folding-upon-binding of an intrinsically disordered protein
Source: Nat Commun. 2025 Nov 27;16:11346. doi: 10.1038/s41467-025-66420-5 (PMC12728182; doi:10.1038/s41467-025-66420-5)
Supplement: Supplementary file 1 — Supplementary Information [file 41467_2025_66420_MOESM1_ESM.pdf]

Supplementary Information

Hierarchical folding-upon-binding of an intrinsically disordered protein

Lenette F. Kjaer, Francesco S. Ielasi, Thomas Winbolt, Elise Delaforge,

Maud Tengo, Luiza Mamigonian Bessa, Laura Mariño Pérez, Elisabetta Boeri Erba,

Guillaume Bouvignies[3]*, Andrés Palencia[2]* and Malene Ringkjøbing Jensen[1]*

* To whom correspondence should be addressed

Dr. Malene Ringkjøbing Jensen, E-mail: malene.jensen@ibs.fr

Dr. Andrés Palencia, E-mail: andres.palencia@inserm.fr

Dr. Guillaume Bouvignies, E-mail: guillaume.bouvignies@ens.psl.eu

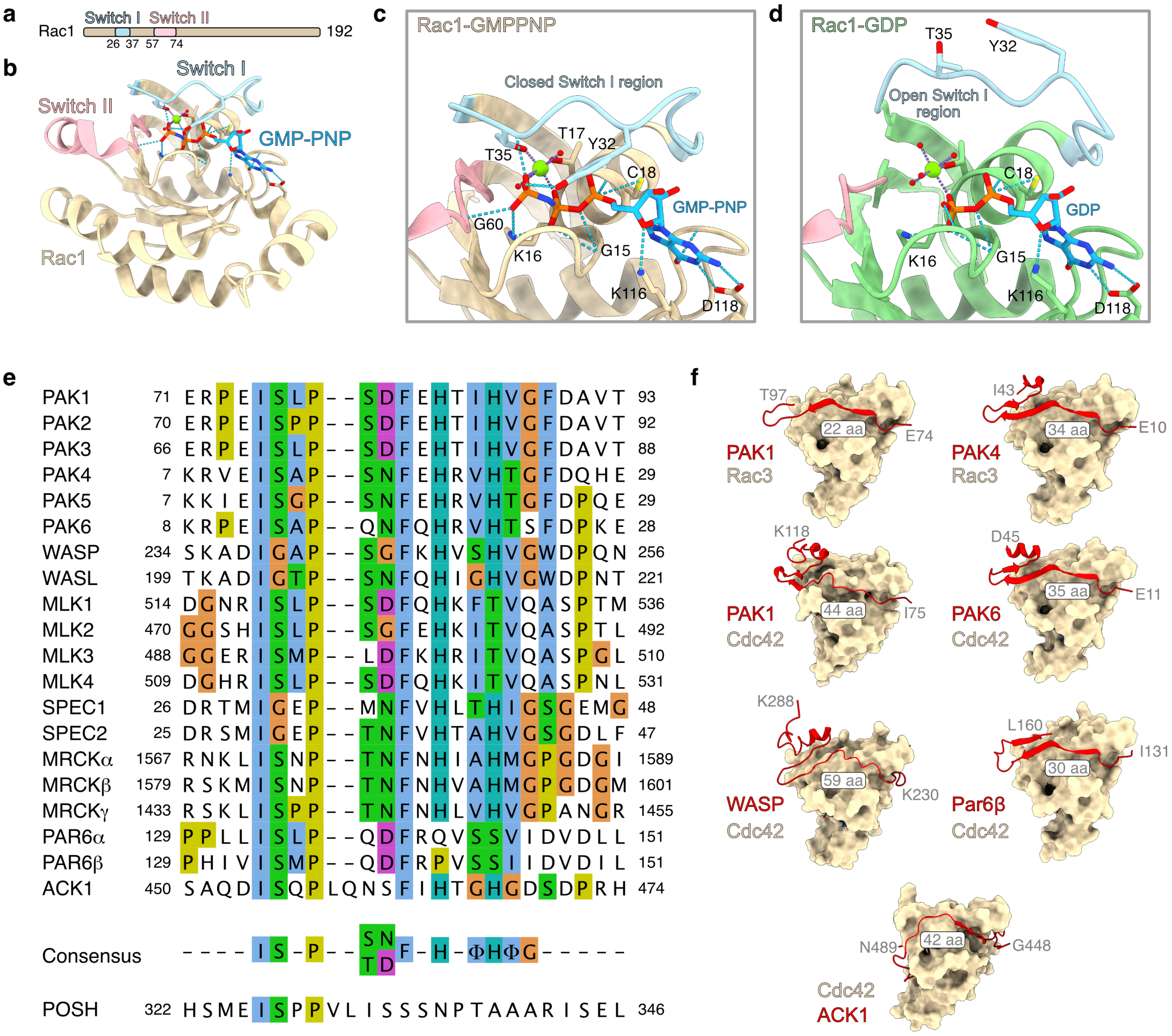

Supplementary Figure 1. Structural features of Rac1 and CRIB motifs. **a**, Domain organization of full-length human Rac1 with the switch I (light blue) and switch II (pink) regions. **b**, Crystal structure of Rac1 in complex with GMPPNP (PDB 3TH5) showing the location of the two switch regions. **c**, Zoom on the structure of Rac1 in complex with GMPPNP (PDB 3TH5) showing the nucleotide binding site. The third phosphate of the GMPPNP makes hydrogen bonds with the backbone amide of T35 and the side chain of Y32 in switch I as well as the backbone amide of G60 in switch II (the Mg^{2+} ion is shown as a green sphere). This leads to a closed switch I loop allowing binding of effectors. **d**, Zoom on the structure of Rac1 in complex with GDP (PDB 5N6O) showing how the switch I loop remains in an open, effector-binding incompatible conformation. **e**, Sequence alignment of known Rac and Cdc42 effectors containing canonical CRIB motifs. The CRIB motif consensus sequence is shown below the sequence alignment with Φ indicating a hydrophobic residue. The consensus sequence is compared to the sequence of POSH showing its categorization as a non-canonical CRIB motif. **f**, Available structures of Rac3 and Cdc42 (beige) in complex with canonical CRIB motifs (red): Rac3-PAK1 (PDB 2QME), Rac3-PAK4 (PDB 2OV2), Cdc42-PAK1 (PDB 1E0A), Cdc42-PAK6 (PDB 2ODB), Cdc42-WASP (PDB 1CEE), Cdc42-Par6β (PDB 1NF3) and Cdc42-ACK1 (PDB 1CF4). For each structure the total number of effector amino acids (aa) in the effector-GTPase complex is indicated.

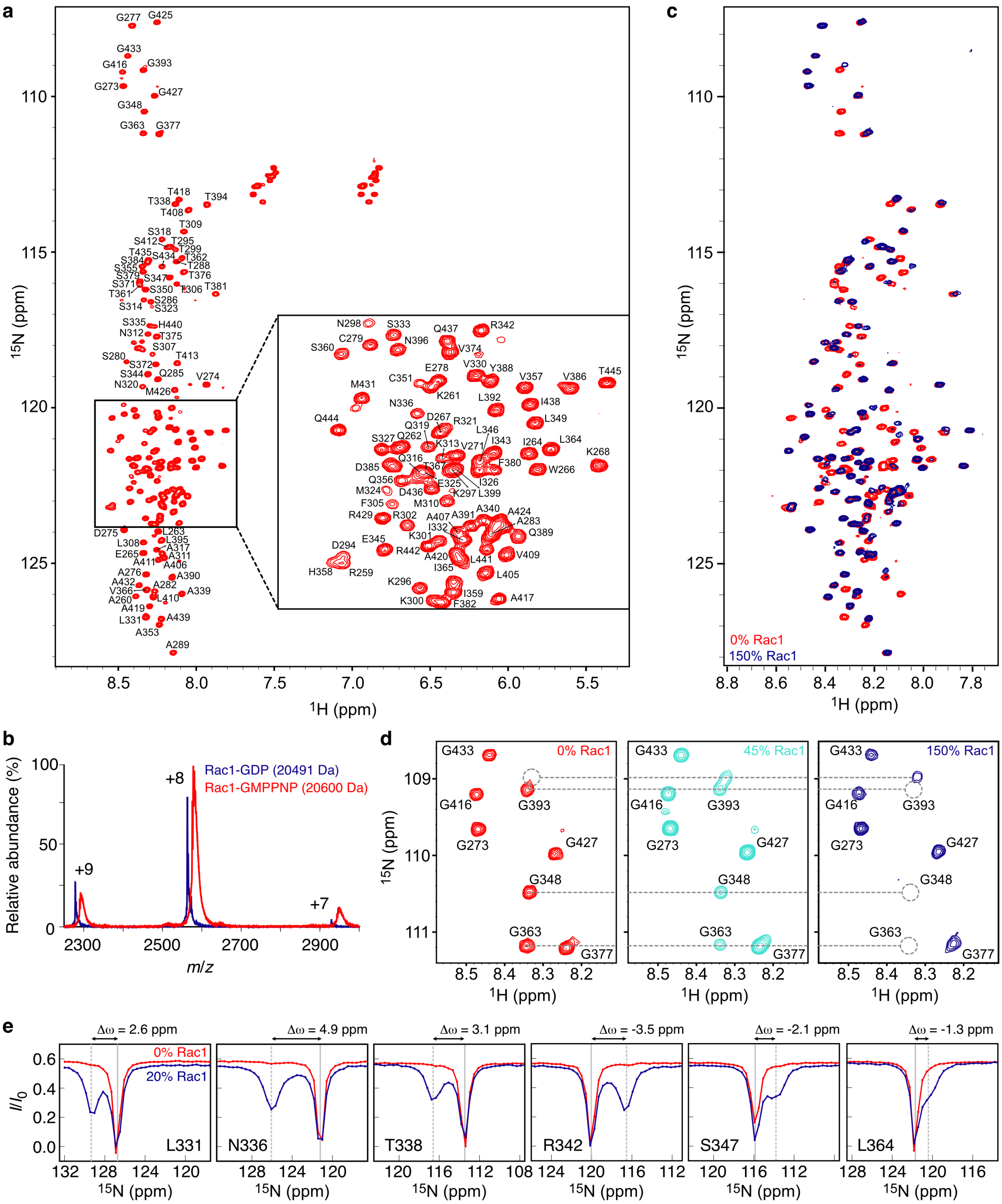

Supplementary Figure 2. Interaction of POSH$_{260\text{-}445}$ with Rac1. a, ^{1}H-^{15}N HSQC spectrum of POSH$_{260\text{-}445}$. The labels correspond to the spectral assignment as obtained from triple resonance experiments. **b**, Native mass spectrometry spectra of Rac1-GDP (blue, before nucleotide exchange, expected mass: 20501 Da) and Rac1-GMPPNP (red, after nucleotide exchange, expected mass with one Mg^{2+} ion: 20601 Da). **c**, ^{1}H-^{15}N HSQC spectrum of POSH$_{260\text{-}445}$ with 0% (red) and 150% (blue) of Rac1 (molar ratio). **d**, Zoom on the glycine-region of the ^{1}H-^{15}N HSQC of POSH$_{260\text{-}445}$ with 0% (red), 45% (cyan) and 150% (blue) Rac1. The resonances of residues located in the direct interaction site with Rac1 disappear and do not reappear upon saturation with Rac1 (see for example G348 and G363). The peaks of residues flanking the direct interaction site split into two resonances upon addition of Rac1, in agreement

with a slow exchange regime (see for example G393). **e**, Selected CEST profiles of $POSH_{260-445}$ with 0% (red) and 20% (blue) Rac1 acquired at 25°C and at a ^{1}H frequency of 700 MHz with a saturation period of 0.4 s and a B_1 field of 21.6 Hz. In the absence of Rac1, no minor dips are observed showing that slow conformational exchange processes are absent in free $POSH_{260-445}$. The minor dips appearing upon addition of Rac1 therefore correspond to the Rac1-bound form of $POSH_{260-445}$. The chemical shift differences, $\Delta\omega$, between the free and the bound state (in Fig. 1g) were extracted as the difference between the observed major (gray solid line) and minor dips (gray dashed line).

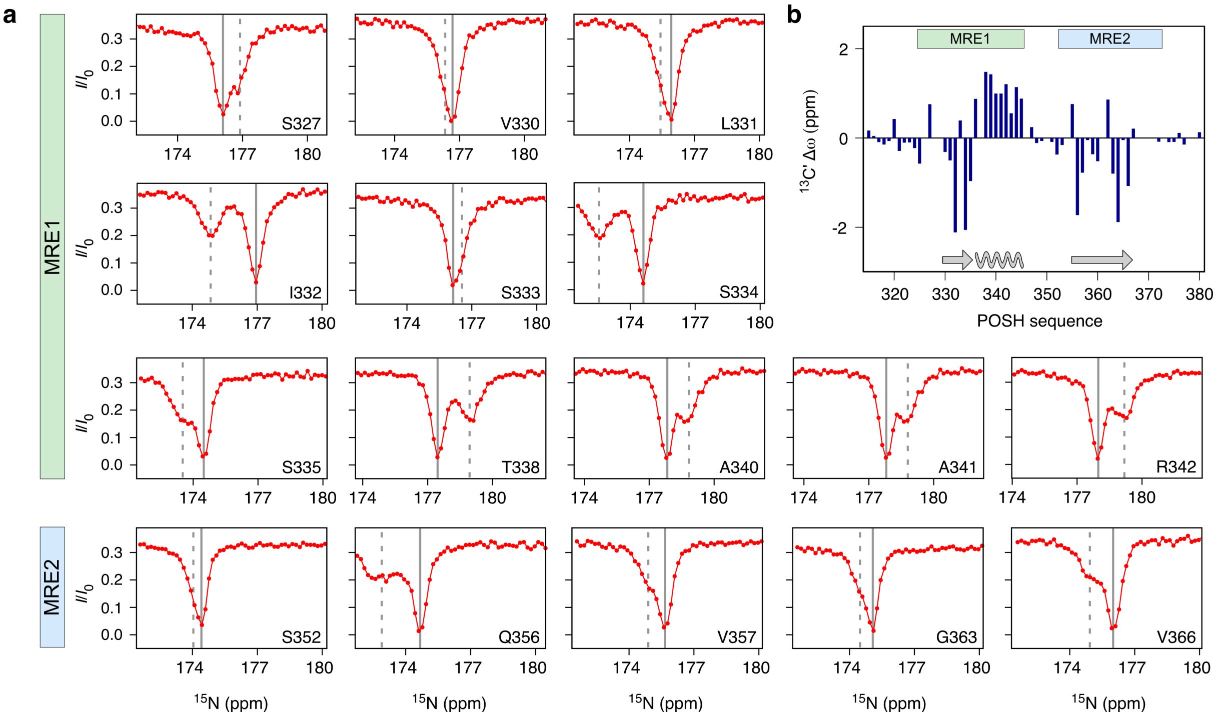

Supplementary Figure 3. ^{13}C′ CEST experiments reveal that POSH adopts secondary structures upon binding to Rac1. **a**, Selected ^{13}C′ CEST profiles across the POSH sequence, covering residues from both MRE1 (top three rows) and MRE2 (bottom row). Experimental data points are plotted as circles, with connecting lines included for visual clarity. The chemical shifts of the major state (free POSH, full-drawn gray line) and the minor state (Rac1-bound POSH, dashed gray line) are indicated. **b**, ^{13}C′ chemical shift differences, Δω, between the free and the Rac1-bound form of POSH, extracted as the difference between the observed major and minor dips in the CEST profiles. The type of secondary structure derived from the ^{13}C′ chemical shifts are shown in gray.

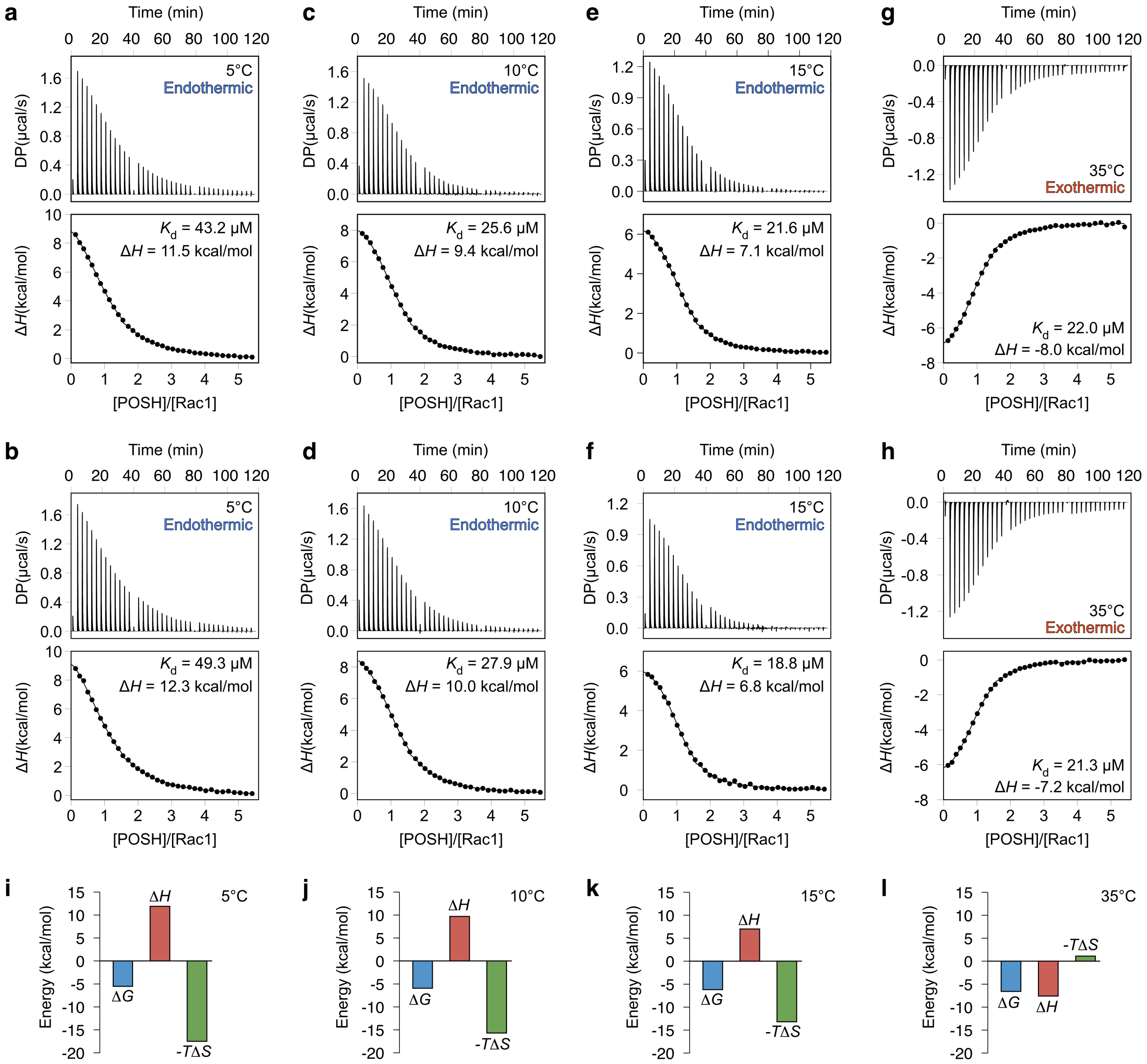

Supplementary Figure 4. ITC titrations of Rac1 with $POSH_{315-380}$. ITC data from three injections were merged by concatenation and acquired in duplicates at 5°C (**a**, **b**), 10°C (**c**, **d**), 15°C (**e**, **f**), and 35°C (**g**, **h**). For all ITC data, raw injection heats are shown on the top and the corresponding specific binding isotherms are shown at the bottom. The data were analyzed according to the binding model "One set of sites" using the PEAQ-ITC analysis software (full-drawn lines). **i**, **j**, **k**, **l**, Dissection of the binding free energies (blue) into enthalpic (red) and entropic (green) contributions at the four temperatures. The average of the duplicate experiments is reported.

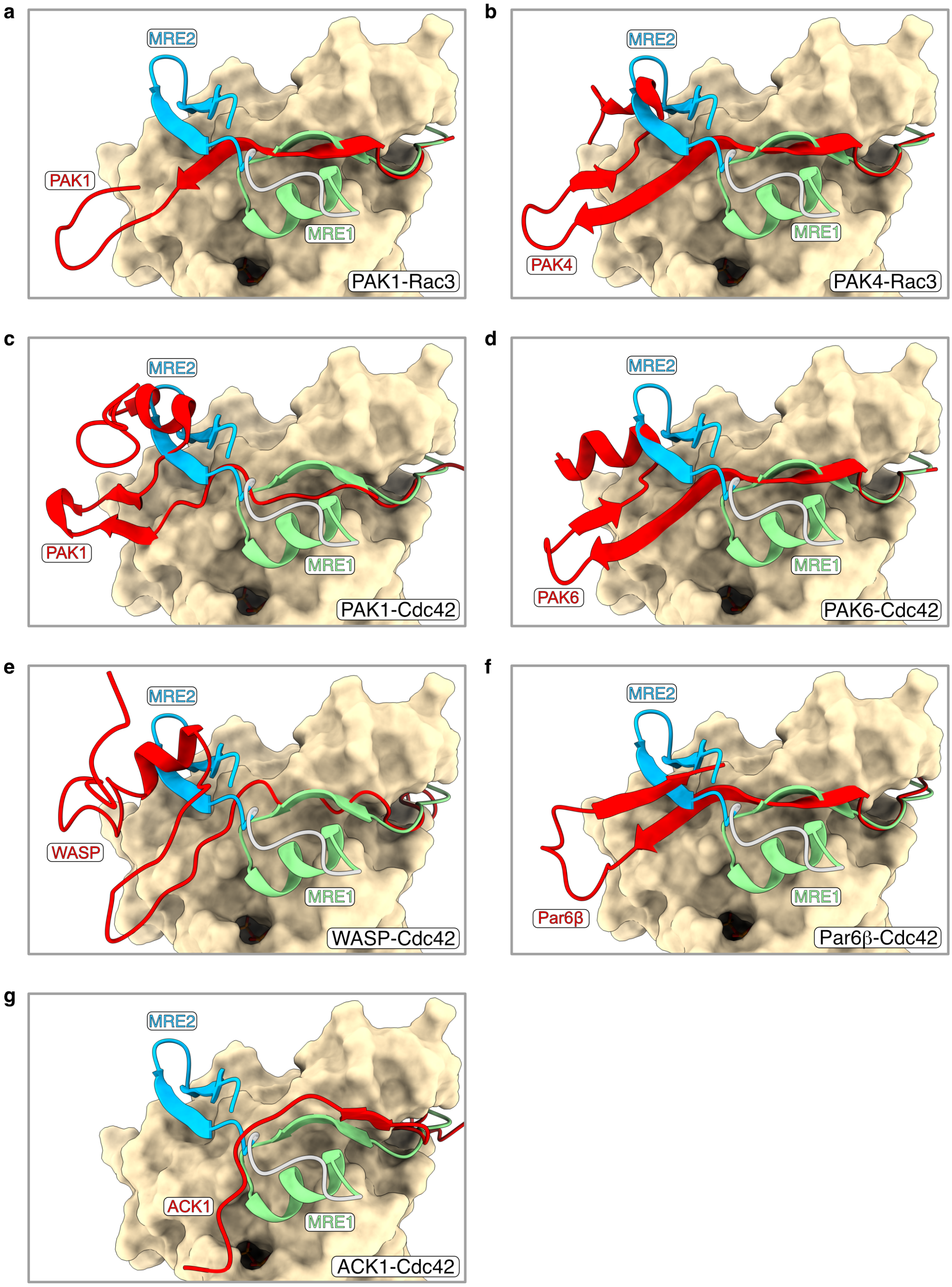

Supplementary Figure 5. Comparison of the GTPase-bound conformation of POSH to that of other signaling effectors. The POSH-Rac1 complex structure (MRE1 - green, linker region - gray, MRE2 - blue) is compared to other solved structures of Rac3 and Cdc42 in complex with the CRIB motifs of signaling effectors (red): **a,** PAK1-Rac3 (PDB 2QME); **b,** PAK4-Rac3 (PDB 2OV2); **c,** PAK1-Cdc42 (PDB 1E0A); **d,** PAK6-Cdc42 (PDB 2ODB); **e,** WASP-Cdc42 (PDB 1CEE); **f,** Par6β-Cdc42 (PDB 1NF3); **g,** ACK1-Cdc42 (PDB 1CF4).

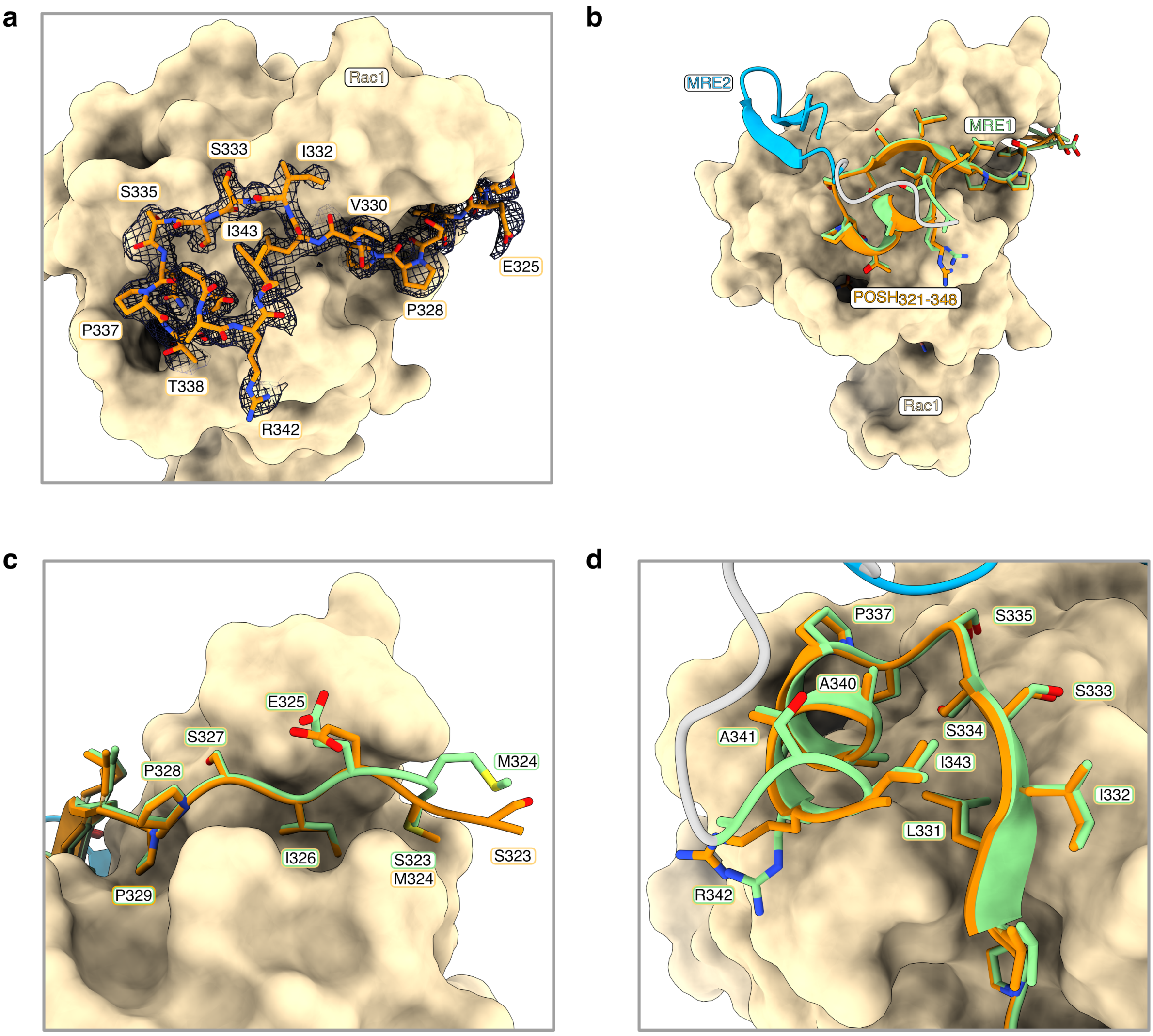

Supplementary Figure 6. Comparison of the crystal structure of the Rac1-POSH$_{321\text{-}348}$ peptide complex with that of the Rac1-POSH$_{319\text{-}371}$ fusion complex. a, Composite omit electron density map of POSH$_{321\text{-}348}$ (orange) contoured at 1.1σ. Rac1 is shown as a beige surface. **b,** Superposition of the crystal structure of the Rac1-POSH$_{321\text{-}348}$ peptide complex (orange) and the Rac1-POSH$_{319\text{-}371}$ fusion complex (MRE1 - green, linker region - gray, MRE2 - blue). **c**, Zoom on the partial CRIB motif of MRE1 in the two structures shown in panel **b**. The side chain conformations are highly similar between the two structures, except for S323 and M324. **d**, Zoom on the β-strand and α-helical motif of MRE1 in the two structures shown in panel **b**. All side chain conformations are conserved between the peptide and fusion complex, except for R342.

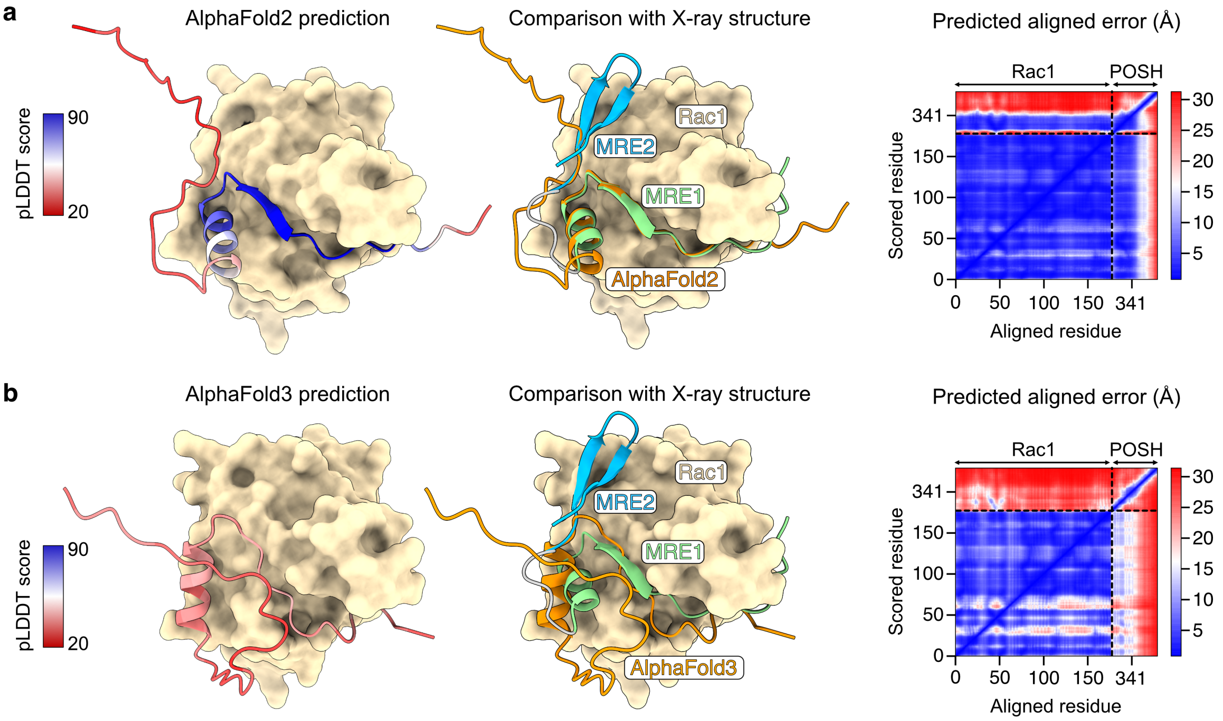

Supplementary Figure 7. AlphaFold structural predictions of the POSH-Rac1 complex. **a,** (Left) AlphaFold2 prediction of the POSH-Rac1 complex with POSH shown in cartoon representation colored according to the predicted local distance difference test (pLDDT) score. (Middle) Superposition of the AlphaFold2 model (orange) on the crystal structure (blue, gray, and green). (Right) Predicted aligned error (PAE) of the AlphaFold2 prediction. **b,** The same as in panel **a**, except AlphaFold3 was used for the structural prediction.

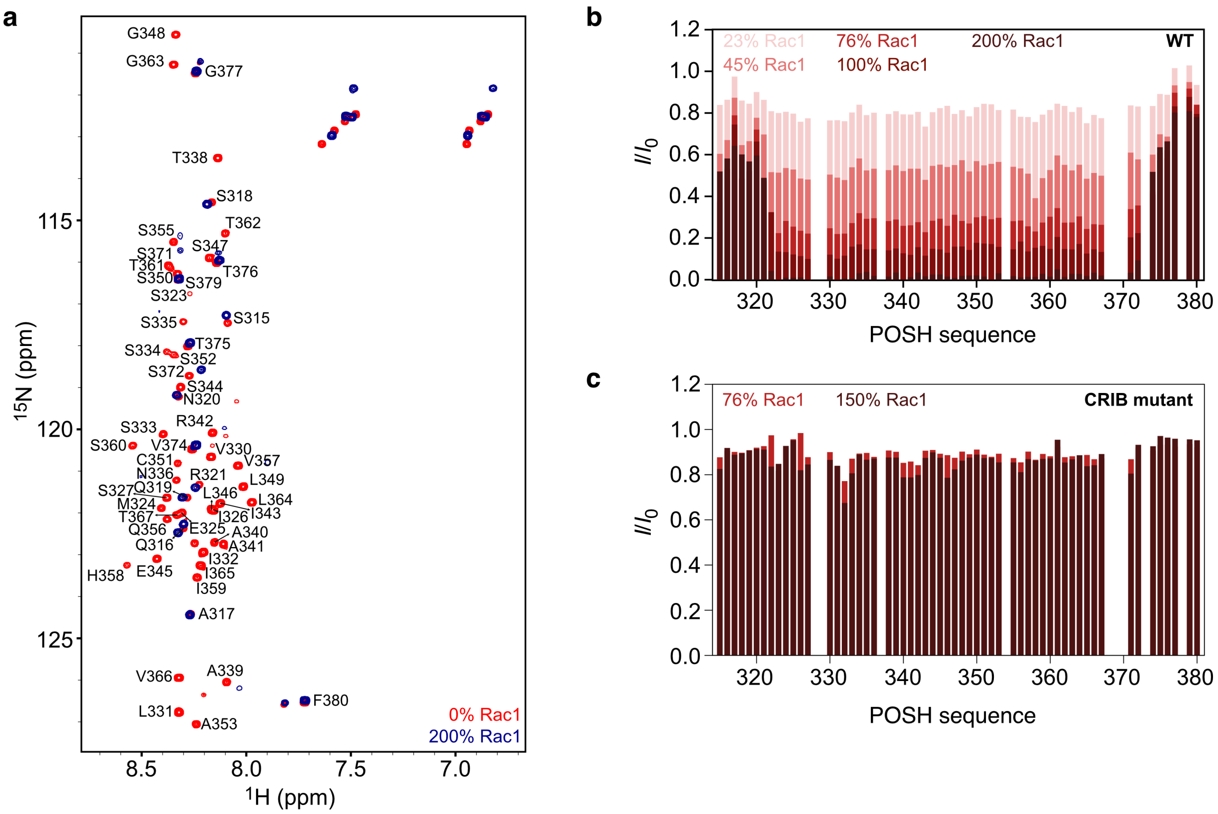

Supplementary Figure 8. Interaction of POSH$_{315\text{-}380}$ with Rac1. a, Assigned ^{1}H-^{15}N HSQC spectra of POSH$_{315\text{-}380}$ recorded at 25°C in the absence (red) and presence (blue) of a 200% molar ratio of Rac1. **b**, Intensity ratios (I/I_0) from titration of ^{15}N-labeled POSH$_{315\text{-}380}$ with Rac1. I_0 represents the peak intensities in the absence of Rac1, while I corresponds to the intensities at increasing Rac1 molar ratios: 23%, 45%, 76%, 100% and 200% (light red to dark red). Progressive decreases in signal intensities prove a direct interaction between POSH$_{315\text{-}380}$ and Rac1. **c**, Intensity ratios (I/I_0) from titration of the CRIB mutant (^{326}ISPP^{329} → ^{326}RRPR^{329}) of ^{15}N-labeled POSH$_{315\text{-}380}$ with Rac1. Ratios are shown for 76% (red) and 150% (dark red) Rac1 molar ratios. No significant changes in signal intensities are observed, showing that the CRIB mutant of POSH does not interact with Rac1.

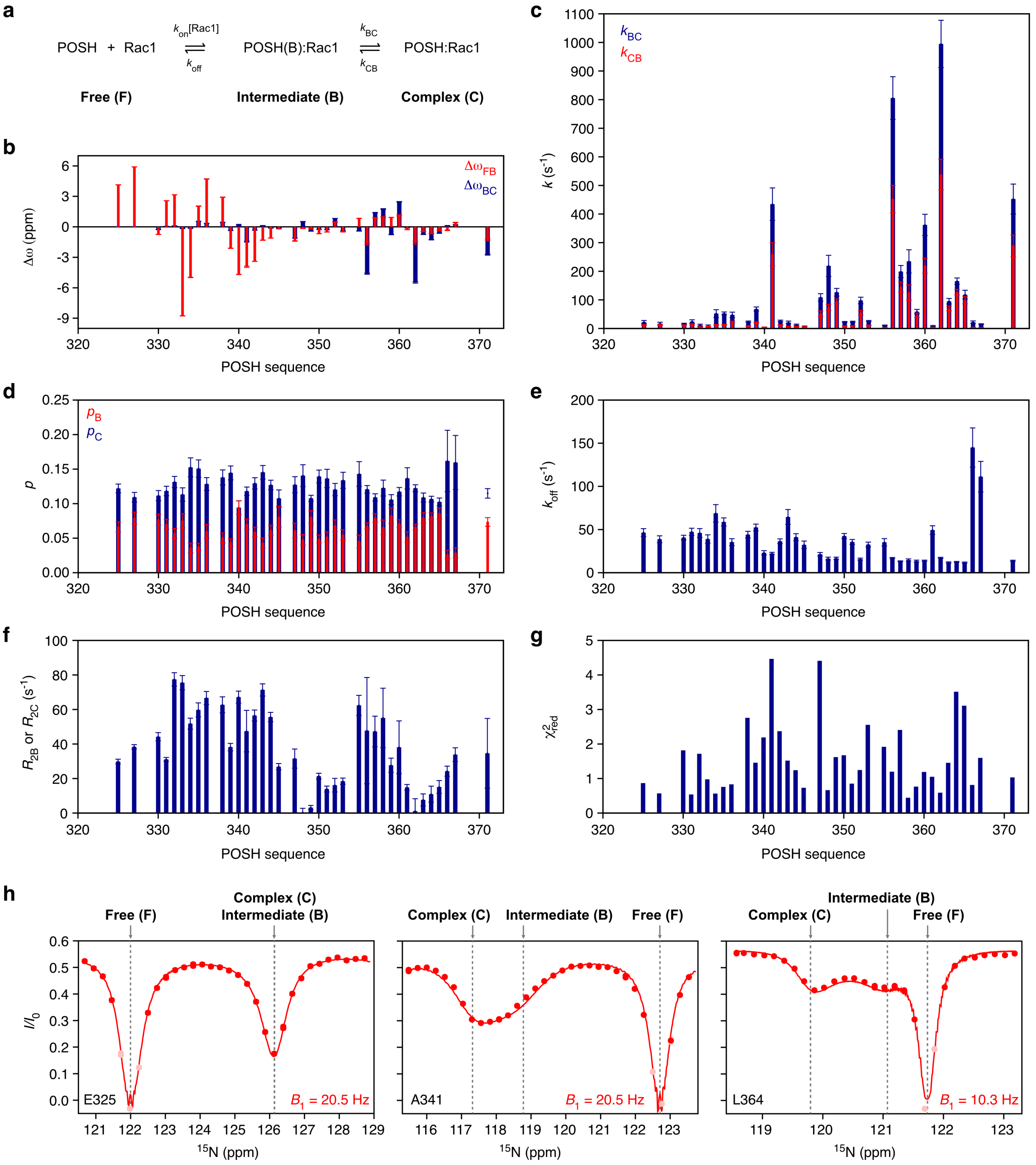

Supplementary Figure 9. Single-residue analysis of ^{15}N CEST data using a 3-site exchange model. **a**, Schematic representation of the 3-site exchange model used for analyzing the ^{15}N CEST data. **b-g**, Results of the analysis of the ^{15}N CEST data of POSH in the presence of Rac1 at a 20% molar ratio, across multiple B_1 saturating fields (5.2, 10.3, 20.5 and 68.6 Hz), using the 3-site exchange model. The analysis was performed separately for each residue in POSH assuming $R_{2B} = R_{2C}$ and a complex dissociation constant of $K_d = 24$ μM. The extracted exchange parameters for each residue in POSH are presented as follows: **b,** Chemical shift differences between states F and B ($\Delta\omega_{FB}$, in red) and between states B and C ($\Delta\omega_{BC}$, in blue), **c,** The forward (k_{BC}, in blue) and backward (k_{CB}, in red) rate constants for the interconversion between the states B and C. **d,** Population of state B (p_B, in red) and state C (p_C, in blue). **e,** Dissociation rate constant, k_{off}. **f,** ^{15}N transverse relaxation rates, R_2, of state B and C (assumed equal). **g,** Per-residue reduced χ^2 values from the 3-site exchange model analysis. **h,** Examples of

experimental ^{15}N CEST data (red and pink circles) for three different residues: E325 (left, B_1 = 20.5 Hz), A341 (middle, B_1 = 20.5 Hz), and L364 (right, B_1 = 10.3 Hz). The data were analyzed for each residue separately across multiple B_1 saturating fields (5.2, 10.3, 20.5 and 68.6 Hz) according to the 3-site exchange model (red line). The minor dips observed in the CEST profiles were assigned either to state B or state C on the basis of their populations (see panel **d**). Data points shown in pink were excluded from the analysis (see Methods). In all panels, error bars represent uncertainties estimated from the covariance matrix of the Levenberg-Marquardt minimization.

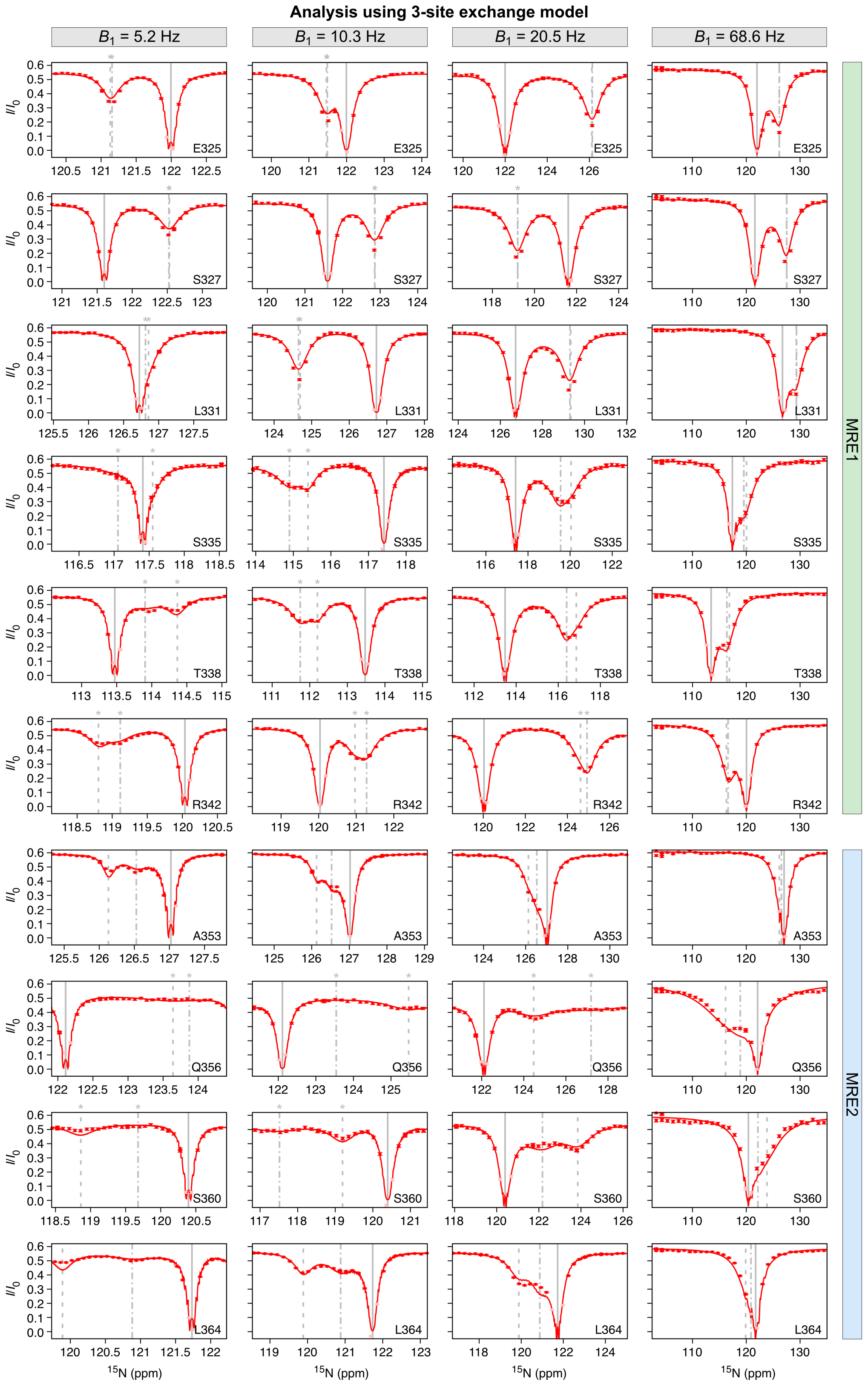

Supplementary Figure 10. Global analysis of ^{15}N CEST data of POSH with a 20% molar ratio of Rac1 using a 3-site exchange model. Selected CEST profiles of residues in both MRE1 and MRE2, measured at four different B_1 field strengths. Experimental data (red circles) were globally analyzed using a 3-site exchange model across all residues in POSH and all B_1 fields (red lines). Vertical gray lines represent the chemical shifts of the free state of POSH

(solid line), the intermediate B (dash-dotted line) and the final bound complex (dashed line). Gray stars indicate resonances that are aliased into the spectral window. Data points shown in pink were excluded from the analysis (see Methods). Error bars represent uncertainties estimated from the noise variance in the CEST profiles (see Methods).

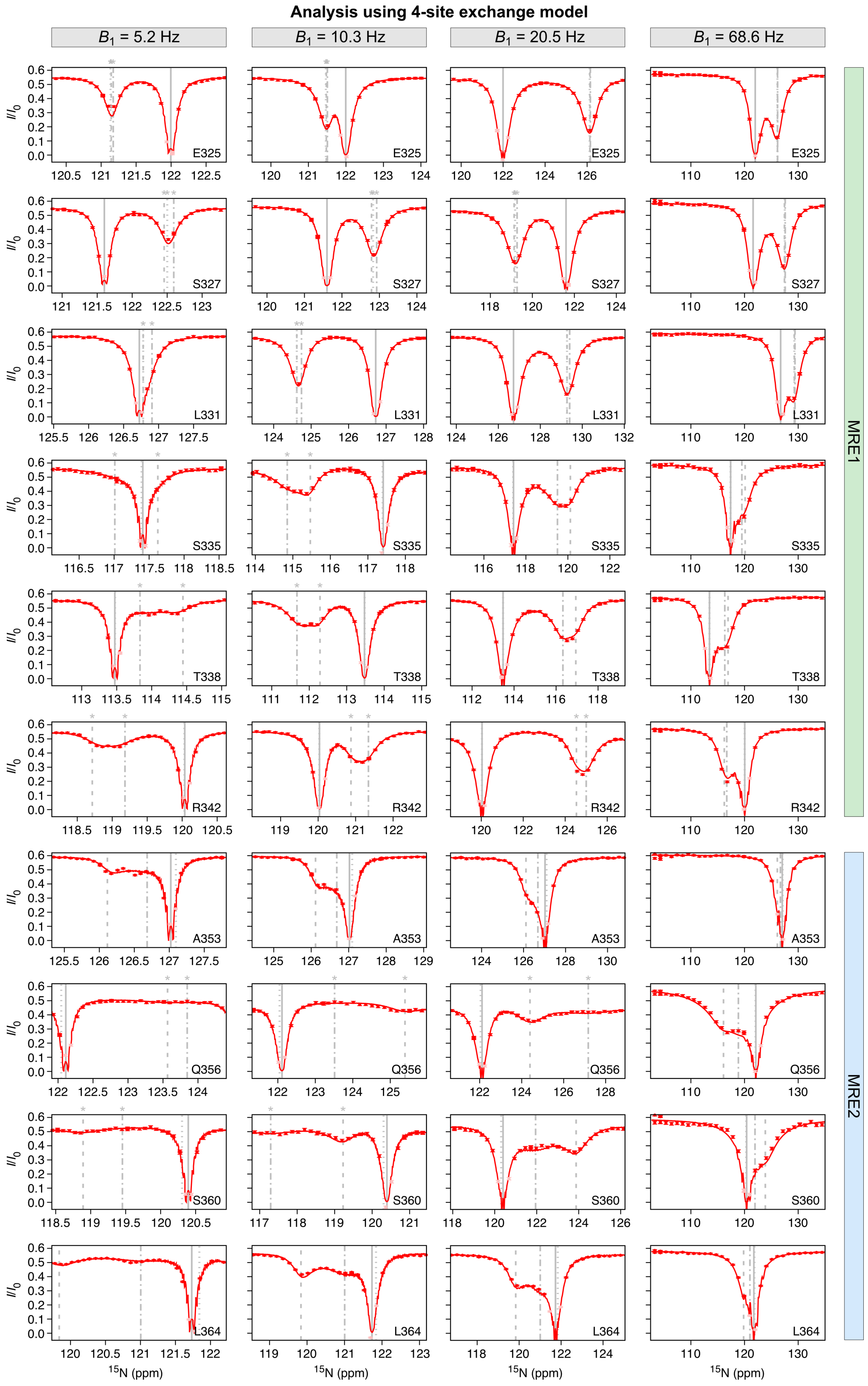

Supplementary Figure 11. Global analysis of ^{15}N CEST data of POSH with a 20% molar ratio of Rac1 using a 4-site exchange model. Selected CEST profiles of residues in both MRE1 and MRE2, measured at four different B_1 field strengths. Experimental data (red circles) were globally analyzed using a 4-site exchange model across all residues in POSH and all B_1 fields (red lines). Vertical gray lines represent the chemical shifts of the free state of POSH

(solid line), the intermediate A (dotted line), the intermediate B (dash-dotted line) and the final bound complex (dashed line). Gray stars indicate resonances that are aliased into the spectral window. Data points shown in pink were excluded from the analysis (see Methods). Error bars represent uncertainties estimated from the noise variance in the CEST profiles (see Methods).

a

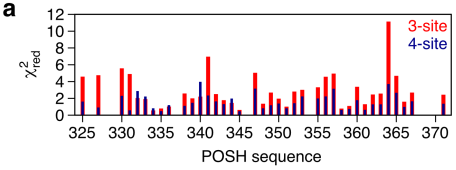

b

Model	χ^2	Number of data points	Number of parameters	Significance
3-site (null)	14850	5295	220	-
4-site (alternative)	8288	5295	260	p < 0.0001

c

d

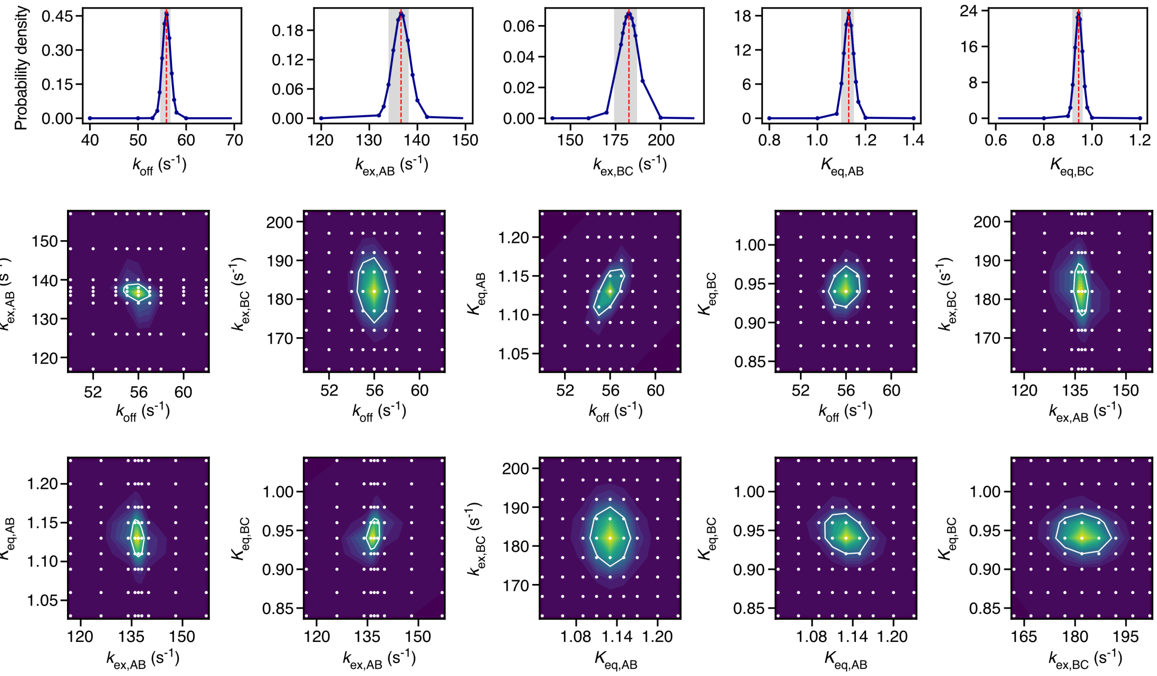

Supplementary Figure 12. Statistics and grid searches from the analysis of the ^{15}N CEST data using 3- and 4-site exchange models. **a**, Per-residue reduced χ^2 obtained from the global analysis of the ^{15}N CEST data across all residues and for all B_1 field strengths using the 3-site model (red) or 4-site model (blue). **b**, Standard F-test confirming that the 4-site model (alternative) provides a statistically significant improvement in data fitting relatively to the 3-site model (null). **c**, One-dimensional grid searches showing normalized probability density distributions for individual global exchange parameters in the 4-site model, based on fits to the ^{15}N CEST data. In these searches, the parameter of interest was fixed at a series of values, while all other parameters were optimized using the same fitting procedure as in the regular 4-site model analysis. Probability densities were computed by transforming the resulting χ^2 landscape into likelihood space and normalizing. Vertical dashed lines indicate best-fit values; shaded regions represent 68% confidence intervals. **d**, Two-dimensional grid searches over parameter pairs in the 4-site model, showing normalized probability density surfaces. White contours enclose 68% confidence regions, and white dots mark sampled parameter combinations. The color scale ranges from dark blue (zero probability density) to yellow (highest). Probability densities were computed by transforming the χ^2 landscape into likelihood space and normalizing.

Supplementary Table 1. Thermodynamic parameters derived from ITC titrations of GMPPNP-loaded Rac1 with $POSH_{315-380}$ at four different temperatures. Errors bars on the average (Avg) correspond to the standard deviation from the duplicate measurements (technical replicates).

Temperature (°C)		Number of sites	K_D (μM)	ΔH (kcal/mol)	$-T\Delta S$ (kcal/mol)	ΔG (kcal/mol)
5		1.13	43.2	11.5	-17.1	-5.56
		1.13	49.3	12.3	-17.8	-5.48
	Avg	1.13 ± 0.01	46.3 ± 4.3	11.9 ± 0.6	-17.5 ± 0.5	-5.52 ± 0.06
10		1.17	25.6	9.4	-15.4	-5.95
		1.24	27.9	10.0	-15.9	-5.90
	Avg	1.21 ± 0.05	26.8 ± 1.6	9.7 ± 0.4	-15.7 ± 0.4	-5.93 ± 0.04
15		1.13	18.8	6.8	-13.0	-6.23
		1.16	21.6	7.1	-13.3	-6.15
	Avg	1.15 ± 0.02	20.2 ± 2.0	7.0 ± 0.2	-13.2 ± 0.2	-6.19 ± 0.06
35		1.04	22.0	-8.0	1.5	-6.57
		1.03	21.3	-7.2	0.6	-6.58
	Avg	1.04 ± 0.01	21.7 ± 0.5	-7.6 ± 0.6	1.1 ± 0.6	-6.58 ± 0.01

Supplementary Table 2. Data collection and refinement statistics for the Rac1-POSH crystal structures.

	**Rac1-POSH$_{321-348}$ peptide complex**	**Rac1-POSH$_{319-371}$ fusion complex**
**Data collection**		
Beamline	ID30A-1 /MASSIF-1	ID30A-1 /MASSIF-1
Date	21/07/2022	13/11/2022
Wavelength	0.9655	0.9655
Space group	$P4_32_12$	$P2_12_12_1$
Cell dimensions		
a, b, c (Å)	54.793, 4.793, 329.942	39.222, 3.082, 73.283
α, β, γ (°)	90.00, 90.00, 90.00	90.00, 90.00, 90.00
Resolution (Å)	19.67-1.85 (2.03-1.85)	51.74-1.24 (1.34-1.24)
R_{merge}	0.18 (1.19)	0.09 (1.68)
I / σI	8 (1.7)	14.9 (1.4)
Completeness - ellipsoidal (%)	94.2 (66.4)	95.0 (55.7)
Multiplicity	13.4 (10.1)	12.9 (11.8)
CC (1/2)	99.7 (81.2)	99.9 (57.6)
**Refinement**		
Resolution (Å)	1.85	1.25
No. reflections work/free	32434/1624	47575/2473
R_{work}-R_{free}	0.195-0.226	0.134-0.164
No. atoms Overall	3571	1958
Protein	3108	1724
Ligand (GMPPNP)	60	32
Water/Others	403	203
B-factors		
Overall	27.1	19.0
Protein	25.8	16.9
Ligand (GMPPNP)	21.4	13.1
Water/Others	38.5	31.6
R.M.S. deviations		
Bond lengths (Å)	0.0084	0.0120
Bond angles (°)	1.4119	1.7831
Rama Favored (%)	92.7	98.0
Rama Outliers (%)	0	0

Note: Data in parentheses are for the highest resolution shell.

Supplementary Table 3. Exchange parameters derived from ^{15}N CEST data of POSH with a 20% molar ratio of Rac1. The parameters were obtained from a global analysis across all POSH residues and B_1 fields, using either a 3-site or a 4-site exchange model. Errors represent 68% confidence intervals derived from a bootstrap analysis consisting of 300 iterations (see Methods).

	**3-site model**	**4-site model**
χ^2_{red}	2.9	1.6
k_{on} (×10^5 M^{-1}s^{-1})	5.1 ± 0.1	7.3 ± 0.2
k_{off} (s^{-1})	44 ± 3	56 ± 2
k_{AB} (s^{-1})	-	72 ± 3
k_{BA} (s^{-1})	-	64 ± 3
k_{BC} (s^{-1})	22 ± 2	89 ± 8
k_{CB} (s^{-1})	8.5 ± 0.7	94 ± 9
p_{A} (%)	-	5.9 ± 0.2
p_{B} (%)	5.3 ± 0.2	6.7 ± 0.2
p_{C} (%)	13.6 ± 0.2	6.3 ± 0.2
