# Supplementary material for: Hierarchical folding-upon-binding of an intrinsically disordered protein
Source: Nat Commun. 2025 Nov 27;16:11346. doi: 10.1038/s41467-025-66420-5 (PMC12728182; doi:10.1038/s41467-025-66420-5)
Supplement: Supplementary file 2 — Description of Additional Supplementary Files [file 41467_2025_66420_MOESM2_ESM.pdf]

Description of Additional Supplementary Files

File Name: Supplementary Movie 1
Description: Hierarchical folding of the scaffold protein POSH upon binding to the small GTPase Rac1. This animation illustrates the hierarchical folding trajectory of the scaffold protein POSH as it interacts with the small GTPase Rac1. In its unbound state, POSH exists as a dynamic ensemble of random coil conformations. Binding is initiated through the engagement of the partial CRIB motif within MRE1, which anchors POSH to Rac1 promoting the formation of a short βstrand while the remainder of the protein remains disordered (Intermediate A). This is followed by folding of the remaining portion of MRE1 and a conformational collapse of MRE2 onto the Rac1 surface, where it explores binding-competent conformations. In the final step, the β-hairpin of POSH folds completely resulting in the fully bound and structured complex. The animation was generated using Chimera (UCSF).
